# Oxygen drives hepatocyte differentiation and phenotype stability in liver cell lines

**DOI:** 10.1007/s12079-018-0456-4

**Published:** 2018-02-04

**Authors:** Martien van Wenum, Aziza A. A. Adam, Vincent A. van der Mark, Jung-Chin Chang, Manon E. Wildenberg, Erik J. Hendriks, Aldo Jongejan, Perry D. Moerland, Thomas M. van Gulik, Ronald P. Oude Elferink, Robert A. F. M. Chamuleau, Ruurdtje Hoekstra

**Affiliations:** 1 Tytgat Institute for Liver and Intestinal Research, Academic Medical Center (AMC), Meibergdreef 69-71, 1105BK Amsterdam, The Netherlands; 2 Surgical Laboratory, Department of Surgery, Academic Medical Center, Meibergdreef 9, 1105AZ Amsterdam, The Netherlands; 3 Bioinformatics Laboratory, Department of Clinical Epidemiology, Biostatistics and Bioinformatics, Academic Medical Center, Meibergdreef 9, 1105AZ Amsterdam, The Netherlands

**Keywords:** HepaRG, C3A, Hepatocyte differentiation, Oxygen, Hepatic progenitor cell, Propagation capacity

## Abstract

The *in vitro* generation of terminally differentiated hepatocytes is an unmet need. We investigated the contribution of oxygen concentration to differentiation in human liver cell lines HepaRG and C3A. HepaRG cells were cultured under hypoxia (5%O_2_), normoxia (21%O_2_) or hyperoxia (40%O_2_). Cultures were analysed for hepatic functions, gene transcript levels, and protein expression of albumin, hepatic transcription factor CEBPα, hepatic progenitor marker SOX9, and hypoxia inducible factor (HIF)1α. C3A cells were analysed after exposure to normoxia or hyperoxia. In hyperoxic HepaRG cultures, urea cycle activity, bile acid synthesis, CytochromeP450 3A4 (*CYP3A4*) activity and ammonia elimination were 165–266% increased. These effects were reproduced in C3A cells. Whole transcriptome analysis of HepaRG cells revealed that 240 (of 23.223) probes were differentially expressed under hyperoxia, with an overrepresentation of genes involved in hepatic differentiation, metabolism and extracellular signalling. Under hypoxia, CYP3A4 activity and ammonia elimination were inhibited almost completely and 5/5 tested hepatic genes and 2/3 tested hepatic transcription factor genes were downregulated. Protein expression of SOX9 and HIF1α was strongly positive in hypoxic cultures, variable in normoxic cultures and predominantly negative in hyperoxic cultures. Conversely, albumin and CEBPα expression were highest in hyperoxic cultures. HepaRG cells that were serially passaged under hypoxia maintained their capacity to differentiate under normoxia, in contrast to cells passaged under normoxia. Hyperoxia increases hepatocyte differentiation in HepaRG and C3A cells. In contrast, hypoxia maintains stem cell characteristics and inhibits hepatic differentiation of HepaRG cells, possibly through the activity of HIF1α.

**Electronic supplementary material:**

The online version of this article (10.1007/s12079-018-0456-4) contains supplementary material, which is available to authorized users.

## Introduction

There is a need for terminally differentiated hepatocytes that can be maintained *in vitro*. Continuous efforts in unravelling the processes underlying hepatocyte differentiation, have led to an increased understanding of critical transcription factors (Takayama et al. 2012; Huang et al. 2014), signalling pathways (Boulter et al. 2012; Huch et al. 2015), mechanical forces and paracrine stimuli (Huch et al. 2015), and of ways to influence these *in vitro* through co-culturing (Kidambi et al. 2009; Takebe et al. 2013), culture platforms (van Wenum et al. 2014), small molecules (Huch et al. 2015; Siller et al. 2015) and extracellular matrix constructs (Park et al. 2016; Dunn et al. 1989). However, terminal differentiation *in vitro* remains out of reach, leading to continuation of the search for contributing factors and strategies to improve differentiation grade.

Oxygen concentration is a known morphogen that can direct cell differentiation through factors such as of which the hypoxia-inducible factors (HIFs) (reviewed in (Simon and Keith 2008; Ren et al. 2015)). Little is known about the role of oxygen concentration in hepatocyte differentiation; there are limited data that suggest that atmospheric hypoxia may stimulate hepatic progenitor cell differentiation from embryonic stem cells (Katsuda et al. 2013). Data on the effects of atmospheric hyperoxia on cultured primary hepatocytes are contradicting, some reporting improvement (Kidambi et al. 2009; Poyck et al. 2008; Buck et al. 2014) and other deterioration (Lillegard et al. 2011), of hepatic functions. This may be explained by differences in experimental set-up leading to a difference in oxygen flux at equal starting concentrations, as well as the use of primary hepatocytes, which display biological variability and enter a condition of stress and dedifferentiation after harvesting, leading to significant batch-to-batch variation (Meyer et al. 2013).

HepaRG is a human hepatic progenitor cell line that expresses most progenitor markers and has the capability to reproducibly differentiate into highly functional hepatocyte-like cells (van Wenum et al. 2014; Cerec et al. 2007). These cells acquire a proliferative progenitor phenotype when plated subconfluently, and, after reaching confluence, differentiate into islets of hepatocyte-like cells, surrounded by cholangiocyte-like cells (Gripon et al. 2002).

The phenotype of HepaRG cells remains stable for ~20 passages, after which they lose their ability to differentiate (Laurent et al. 2013). HepaRG cells represent primary hepatocytes (Gao and Liu 2017) to high extent and were therefore selected to study the effects of oxygen on hepatocyte differentiation.

In this study we show that ambient hyperoxia drives HepaRG hepatocyte differentiation, and suggest this might be a general finding for human hepatocyte cell lines by showing the same phenomenon with the human liver cell line C3A. We also show that hypoxia maintains HepaRG cells in a progenitor state and increases their stability.

## Materials and methods

### Cells and culture procedure

Primary human hepatocytes (PHHs) were isolated from the healthy parenchyma in liver resection material from three patients, aged 40, 68 and 70, with liver adenomas or colorectal cancer metastases and no macroscopic signs of liver damage, by a modified 2-step collagenase perfusion technique as described (Hoekstra et al. 2006). Cells were snap-frozen directly after isolation and kept in liquid nitrogen until RNA isolation. The procedure was in accordance with the ethical standards of the institutional committee on human experimentation (protocol number 03/024) and the Helsinki Declaration of 1975. Ethical approval was obtained from the ethics committee of the Academic Medical Center Amsterdam, and informed consent was obtained from all three patients.

HepaRG cells (Biopredic) were maintained under normoxia at 37 °C as described previously (Laurent et al. 2013). For experiments, cells were plated 1:5 in 6-well plates (for immunofluorescence) or 12-well plates (other experiments) (Corning) and cultured without dimethylsulfoxide. Three gas compositions were applied to the cells: 5% O_2_ (=hypoxic; 5% O_2_, 5%CO_2_ and 90%N_2_), 21% O_2_ (=normoxic; 5%CO_2_, 21% O_2_, 75%N_2_) and 40% O_2_ (=hyperoxic; 40% O_2_, 5%CO_2_, 55%N_2_) (Linde Gas) in gastight incubator chambers at 37 °C. For hypoxic and normoxic culturing, the HepaRG cells were immediately after seeding exposed to these gas compositions and cultured for 4 weeks; for hyperoxic culturing the cells were cultured under normoxic conditions during the first 2 weeks and then transferred to the hyperoxic conditions for the following 2 weeks. After four weeks of culturing under the different gas regimes, RNA was harvested and function tests were performed (2 independent experiments, *n* = 3 per experiment).

To test the stability of the cells at serial passaging, the cultures were split at passage 17 from isolation, and transferred to the normoxic and hypoxic incubator. Cultures were passaged at a regular 1:5 ratio once per two weeks, and for every 2 passage (passage 19, 21 and 23) cells were seeded in 12-well culture plates, cultured under normoxia for 4 weeks and tested for functionality and transcript levels (3 independent experiments, *n* = 3 per experiment).

C3A cells (ATCC, CRL10741) were maintained as described (van Wenum et al. 2016). For experiments, cells were plated 1:10 in 12-well plates (Corning) and kept under normoxia for 7 days until testing (normoxic cultures) or transferred to the hyperoxic incubator after 24 h (hyperoxic cultures) until testing at day 7 (2 independent experiments, *n* = 3 per experiment).

### Function tests

The elimination and/or production of ammonia, lactate, glucose and total bile acids, as well as urea cycle activity were tested as described (Hoekstra et al. 2011). Briefly, cultures were exposed to HepaRG culture medium supplemented with 1 mM N-carbamoyl-l-glutamate, 1.5 mM ^15^NH_4_Cl, 2.27 mM D-galactose, 2 mM L-lactate and 125 μM testosterone (all compounds from Sigma Aldrich), and samples were taken after 0.75, 8 and 24 h and analysed for concentrations of ammonia, lactate, glucose, ^15^N urea and total bile acids. Next, the accumulation or disappearance rates could be calculated. The accumulation of ^15^N–urea was used as a measure for urea cycle activity.

Cytochrome P450 (CYP)3A4 activity was quantified with CYP3A4 P450-Glo™ Assays (Promega) according to the manufacturer’s instructions. For optional CYP3A4 induction, the cultures were pre-exposed to 4 μM rifampicin (Sigma Aldrich) for 3 days, and subsequently washed with fresh culture medium before testing.

After testing, the cells were lysed in 1 mL 0.2 M NaOH and total protein content per well was determined using the Bio-Rad Protein Assay (Bio-Rad) for normalization of the functionality data.

### Oxygen measurement

HepaRG cells were seeded in 24-well culture plates (OxoDish®) with oxygen sensor spots at the bottom of the well and subjected to the different gas regimes, as described above. Oxygen concentration at the bottom of the wells was measured real-time and non-invasively through the transparent bottom of the OxoDish® plates after 2 h equilibration, with and without 4 week old cultures, inside the incubators with different oxygen compositions, using the SDR SensorDish® Reader, which was kindly made available by Applikon Biotechnology (*n* = 24/experiment).

### RNA extraction, qRT-PCR and microarray analysis

Cells were lysed in 600 ml RLT buffer (RNeasy; QIAGEN). RNA was extracted according to manufacturer’s instruction. RT-PCRs were performed using gene-specific RT-primers and a touch-down qPCR protocol as described previously (Hoekstra et al. 2005). Primers and template dilutions are listed in Table S1. For microarray analysis, cRNA, obtained from freshly isolated PHHs and from HepaRG cells cultured under normoxia or hyperoxia (*n* = 3/group) was labelled (cRNA labelling kit for Illumina, Ambion), and hybridized after sample randomization to Illumina HumanHT-12 v4 arrays according to manufacturer’s instructions. Image analysis and extraction of raw expression data was performed with Illumina GenomeStudio v2011.1 Gene Expression software with default settings (no background subtraction and no normalization).

Microarray data were analyzed with Bioconductor packages (v2.12) using the statistical software environment R (v3.0.0). Raw data normalization was performed on the Illumina sample and control probe profiles by normexp-by-control background correction, quantile normalization, and log2 transformation using the limma package (version 3.16.8). The arrayQualityMetrics package (version 3.16.0) was used to assess the quality of the microarray data. Probes with a detection *P*-value >0.05 (non-expressed) on all arrays (16,863 of 47,231 probes) were filtered out. Differential expression was assessed using a moderated t-test using the linear model framework from the *limma* package. Resulting *P*-values were corrected for multiple testing using the Benjamini-Hochberg false discovery rate. Probes were reannotated using the IlluminaHumanv4.db package (version 1.18.0). Upstream regulator analysis was performed using the web-based Ingenuity Pathway Analysis package (QIAGEN). Statistical significance of the overlap between the list of genes from our dataset (non-adj. *P* < 0.01 between normoxia and hyperoxia) and target genes in transcription regulator datasets was calculated using the Fisher’s Exact test. The minimum number of overlapping genes was set to 5.

### Western blotting

Cells were lysed in ice-cold nuclear extraction buffer (420 mM NaCl, 20% (*w*/*v*) glycerol, 5 mM MgCl_2_, 5 mM EGTA, 0.5% Nonidet-P40, 20 mM Tris-HCl, pH 8.0) freshly supplemented with complete EDTA-free protease inhibitor cocktail (Roche), and 1 mM dithiothreitol. Next, the samples were centrifuged at 14,000 g for 10 min at 4 °C. The supernatant was harvested for SDS-PAGE. Sixty micrograms of protein per sample were electrophoresed on an 8% SDS-PAGE, transferred to polyvinylidene difluoride (PVDF) membrane (Invitrogen) by semi-dry blotting and blocked overnight in 5% non-fat milk / PBST (phosphate-buffered saline with 0.05% (*w*/*v*) Tween 20). For immunodetection, the PVDF membrane was incubated with a rabbit polyclonal antibody against HIF1α (Abcam, ab2185) diluted 1:1000 for 1 h at room temperature, washed 3× with TBST (Tris-buffered saline with 0.05% (*w*/*v*) Tween 20), incubated with horseradish peroxidase-conjugated goat anti-rabbit IgG antibody (Bio-rad) for 1 h and washed 3× with TBST. All antibodies were diluted in 5% non-fat milk / PBST. The PVDF membrane was developed with home-made enhanced chemiluminescence reagents (100 mM Tris-HCl pH 8.5, 1.25 mM luminol, 0.2 mM p-coumarin and freshly added 3 mM H_2_O_2_) and detected by ImageQuant LAS 4000 (GE Healthcare Life Sciences). For loading control, the PVDF membrane was stripped and reprobed with horseradish peroxidase-conjugated monoclonal rabbit anti-vinculin antibody (Cell Signaling #18799).

### Immunofluorescent staining

HepaRG monolayers were fixed with 2% formalin (VWR) for 2–5 min at room temperature prior to permeabilization with 0.3% Triton X-100 (Bio-Rad) in ice-cold PBS for 20 min and blocked for 1 h with 10% fetal calf serum in PBS on ice. The cells were incubated with 1:200 diluted primary antibodies in PBS overnight at 4 °C followed by 3× wash with ice-cold PBS, and then incubated with 1:1000 diluted secondary (fluorescent) antibody in PBS for two hours at 4 °C. Finally, the monolayers were washed 3× with ice-cold PBS and mounted with DAPI-containing Vectashield (Vector Laboratories). Imaging was performed on a Leika DCF450 microscope.

Primary antibodies were: Goat anti-human albumin (Bethyl Laboratories), Rabbit anti-human SRY-box 9 (SOX9) (Millipore), Rabbit anti-human HIF1α (Abcam), Goat anti-human CCAAT/enhancer-binding protein alpha (CEBPα) (Santa-Cruz). Fluorescent secondary antibodies: Donkey anti-Rabbit, Alexa Fluor-546 (Invitrogen), Donkey anti-Goat, Alexa Fluor-448 (Molecular Probes), Donkey anti-Goat, Alexa Fluor-546 (Invitrogen). Negative controls were performed under the same experimental conditions, without primary antibodies, and were imaged at the same setting (Fig. S1).

### Statistical analyses

Values are given as mean ± standard deviation. Data were analysed and displayed graphically using Prism 7.02 (GraphPad). Student’s *t*-tests, corrected for multiple testing using the Holm-Sidak method were used when comparing two groups. Two-way ANOVA analysis with Dunnett’s multiple comparison procedure was used when comparing more than two groups. Adjusted *P*-values<0.05 were considered statistically significant.

## Results

### HepaRG cells do not acquire hepatocyte morphology under hypoxic conditions and develop more discrete hepatocyte clusters under hyperoxic conditions

**Fig. 1:**
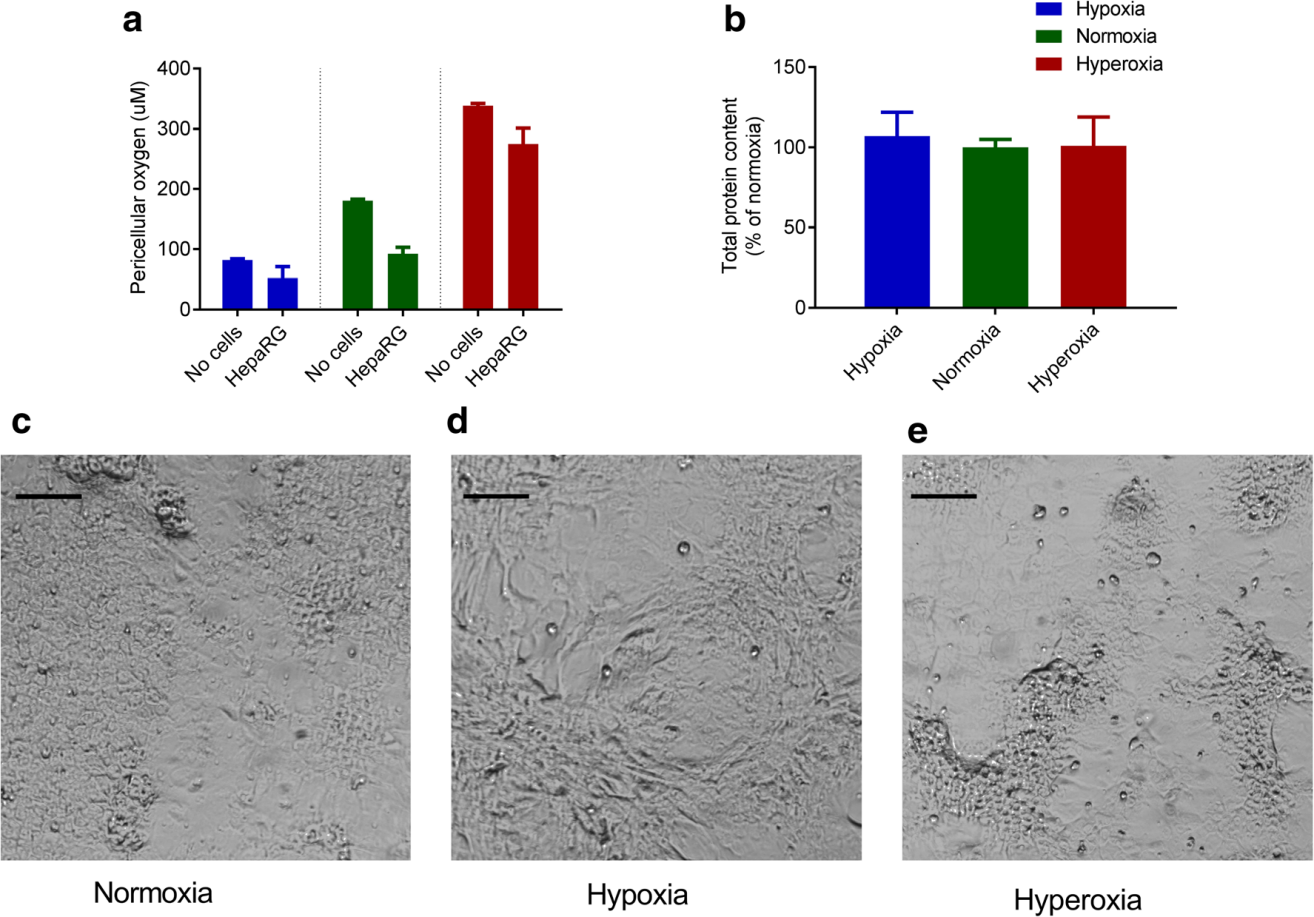
Ambient oxygen concentration influences HepaRG cell morphology but not growth. **a** Pericellular oxygen concentration in culture medium with- and without 4-week old HepaRG cultures and (**b**) total protein content of 4-week old cultures. There was a marked difference in morphology of HepaRG cells cultured under: (**c**) normoxia, (**d**) hypoxia or (**e**) hyperoxia. Scale bar = 100 μM

HepaRG cells were cultured under Normoxia (21% ambient O_2_), Hypoxia (5% ambient O_2_) or Hyperoxia (40% ambient O_2_). The actual oxygen concentration at the bottom of the culture wells was determined in the absence and presence of 4-week-old cultures. The concentrations were 82 ± 1.8, 181 ± 2.4 and 339 ± 3.9 μM O_2_ without cells and 52 ± 19.1, 93 ± 10.4 and 275 ± 26.7 μM O_2_ with cells (Fig. 1a). Total protein analysis revealed no significant difference in proliferation or viability between the cultures (Fig. 1b).

After four weeks in culture, normoxic cultures differentiated into patches of polygonal hepatocytes, surrounded by flat cholangiocyte-like cells, as described (Gripon et al. 2002) (Fig. 1c), while hypoxic cultures consisted of stretched cells without hepatocyte islands (Fig. 1d). When cultures were exposed to ambient hyperoxia from day 1 viability was lost, and therefore cultures in the hyperoxic group were subjected to hyperoxia only after reaching confluence at day 14. After 28 days, the polygonal hepatocyte clusters were more clearly delineated compared to the normoxic cultures (Fig. 1e).

**Fig. 2:**
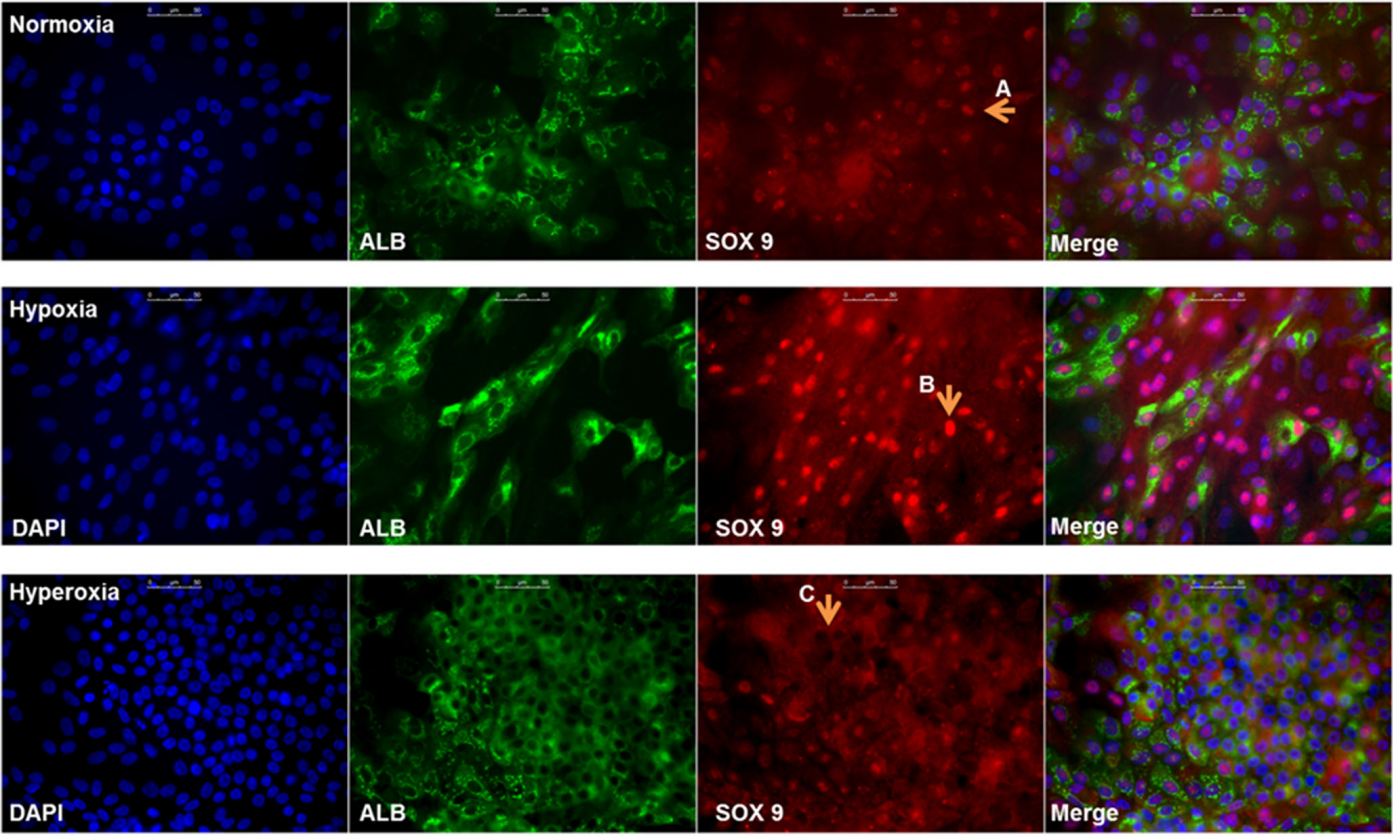

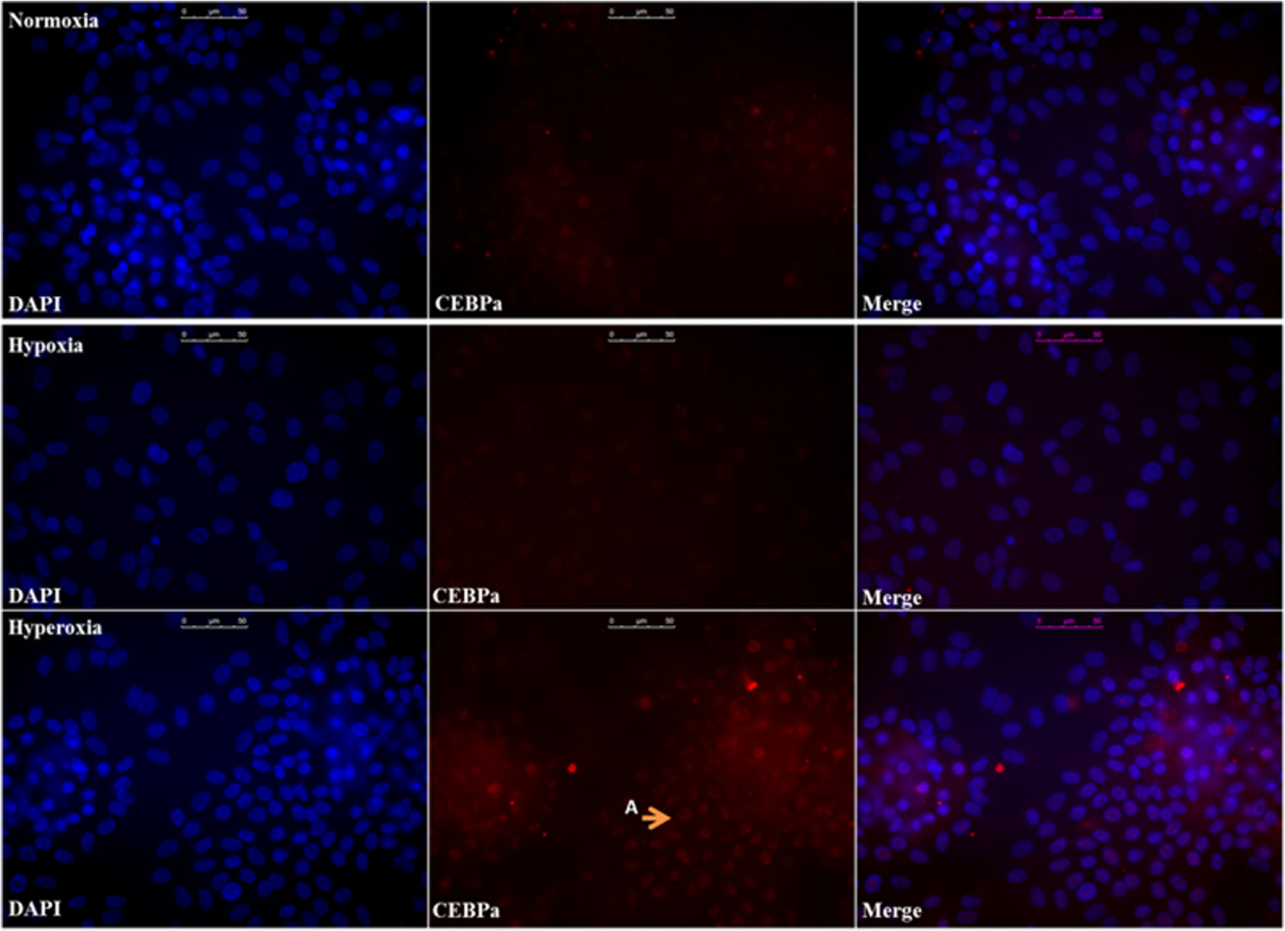
Nuclear SOX9 expression is downregulated, while CEBPα is upregulated at increased oxygen levels. **a** HepaRG cells cultured under normoxia (top), hypoxia (middle) and hyperoxia (bottom), stained for: DAPI (blue), Albumin (green) and SOX9 (red). Arrows A&B indicate nuclear translocation of SOX9 in HepaRG-Hypoxia, and to a lesser degree in HepaRG-Normoxia. Arrow C indicates mainly cytosolic SOX9 expression in HepaRG-Hyperoxia, with SOX9-negative nuclei. **b** HepaRG cells cultured under normoxia (top), hypoxia (middle) and hyperoxia (bottom), stained for: DAPI (blue) and CEBPα (red). Arrow A indicates positive nuclear staining. Scale bar = 50 μM

Protein expression of stem cell marker SOX9 and hepatic transcription factor CEBPα was assessed by immunocytochemistry. A staining was performed for albumin to visualize hepatocyte-like cells (Fig. 2a). Hypoxic cultures were SOX9-positive in most nuclei and partly albumin-positive, while normoxic cultures developed clusters of albumin-positive cells that were partially nuclear SOX9-positive. Hyperoxic cultures formed larger clusters of albumin-positive cells that were in majority SOX9-negative. In contrast, nuclei of hyperoxic cultures were CEBPα-positive, while hypoxic and normoxic cultures were less positive (Fig. 2b).

On the basis of these observations we hypothesized that oxygen is an important factor in determining the differentiation state of HepaRG cells; hypoxia promotes stem cell characteristics, whereas hyperoxia induces hepatic differentiation.

### Hyperoxia augments hepatocyte functionality in HepaRG and C3A cells

**Fig. 3:**
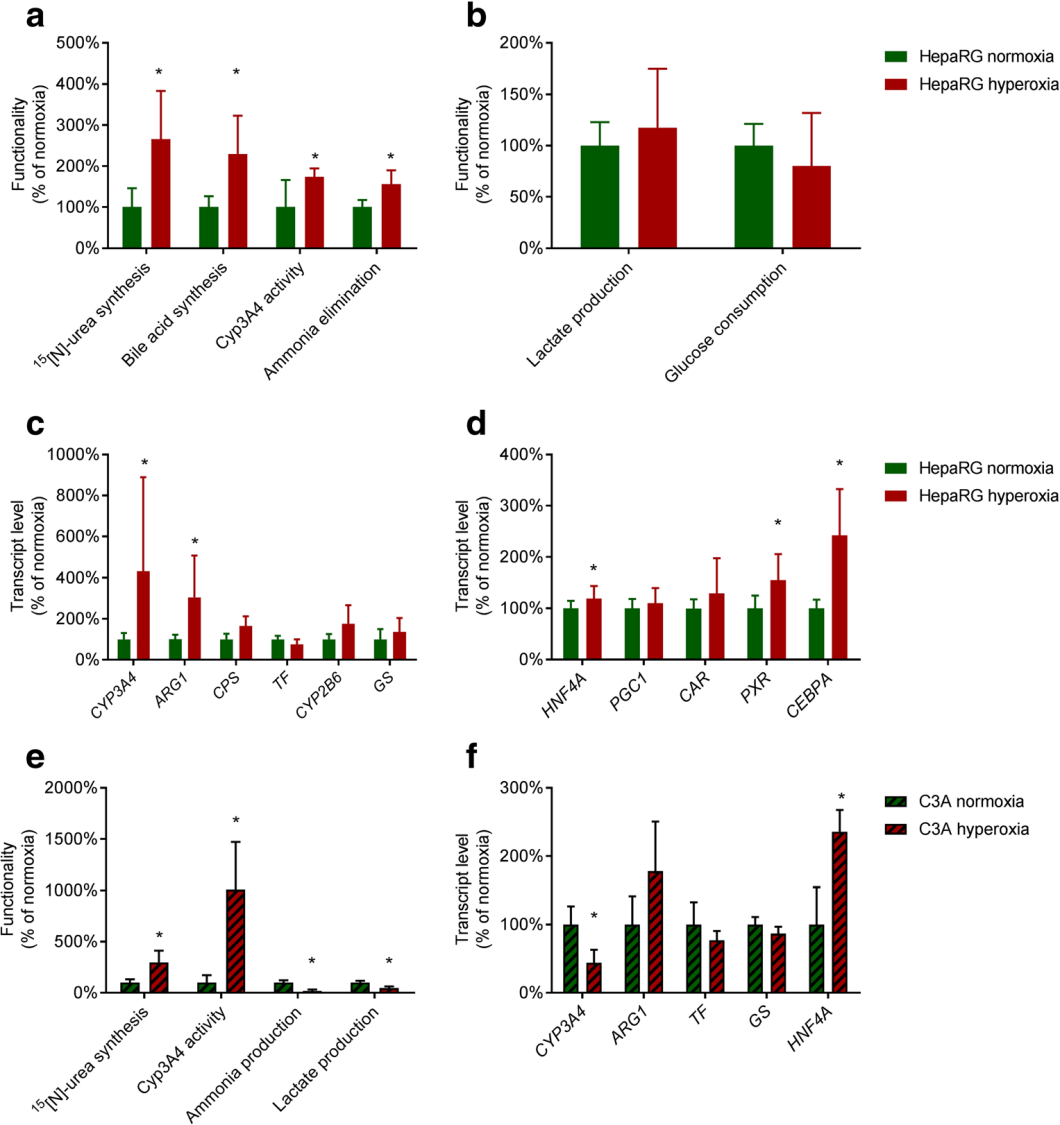
Ambient hypoxia augments hepatic differentiation of HepaRG and C3A cells. HepaRG monolayers cultured under ambient normoxia or hyperoxia were tested for hepatic functions (**a**), glucose consumption and lactate production (**b**), as well as transcript levels of hepatic genes (**c**) and transcription factor genes (**d**). C3A monolayers cultured under normoxia and hyperoxia were tested for hepatic functions (**e**) and hepatic gene transcript levels (**f**). * = *P* < 0.05 compared to HepaRG normoxia

To confirm that hyperoxia increases hepatic differentiation, we analysed hepatic functions and hepatocyte-specific gene transcript levels of HepaRG monolayers cultured under normoxia or hyperoxia. Hyperoxic HepaRG cultures exhibited significantly higher levels of urea cycle activity (266 ± 118%), bile acid synthesis (230 ± 93%), CYP3A4 activity (174 ± 20%) and ammonia elimination (156 ± 34%) compared to normoxic cultures (Fig. 3a), while lactate and glucose metabolism did not differ significantly (Fig. 3b).

Under hyperoxia, transcript levels of the hepatic genes *CYP3A4* and Arginase1 (*ARG1)* were significantly higher (433 ± 457% and 305 ± 203% respectively), while transcript levels of Carbamoyl-phosphate synthase (*CPS1)*, Transferrin (*TF)*, *CYP2B6* and Glutamine synthase (*GS)* were unchanged (Fig. 3c). Transcript levels of hepatic transcription factors Hepatic nuclear factor 4α (*HNF4A)*, Pregnane X receptor (*PXR)* and *CEBPA* were significantly induced at 119 ± 25%, 155 ± 50% and 242 ± 90% respectively, whereas Constitutive androstane receptor (*CAR)* transcript levels were unchanged (Fig. 3d).

To exclude that the effects of ambient hyperoxia are cell-line specific, we repeated the experiments for the hepatoblastoma cell line C3A (Darlington et al. 1987). There was no evident effect on morphology (not shown), however, urea cycle and CYP3A4 activity were induced up to 298 ± 115% and 1008 ± 464%, respectively, compared to normoxic cultures (Fig. 3e). In contrast to HepaRG, C3A cells produce rather than eliminate ammonia (van Wenum et al. 2016). Under hyperoxia, ammonia production was reduced to 19 ± 13% and lactate production to 48 ± 14%. *CYP3A4* transcript levels were significantly reduced (to 44 ± 19%). The transcript levels of *ARG1*, *TF* and *GS* did not differ significantly and *HNF4A* transcript levels were increased (236 ± 32%) (Fig. 3f).

These results confirm that hyperoxia augments hepatic differentiation in hepatic cell lines in general.

### Hyperoxia induces upregulation of transcription factors involved in hepatocyte differentiation.

**Fig. 4:**
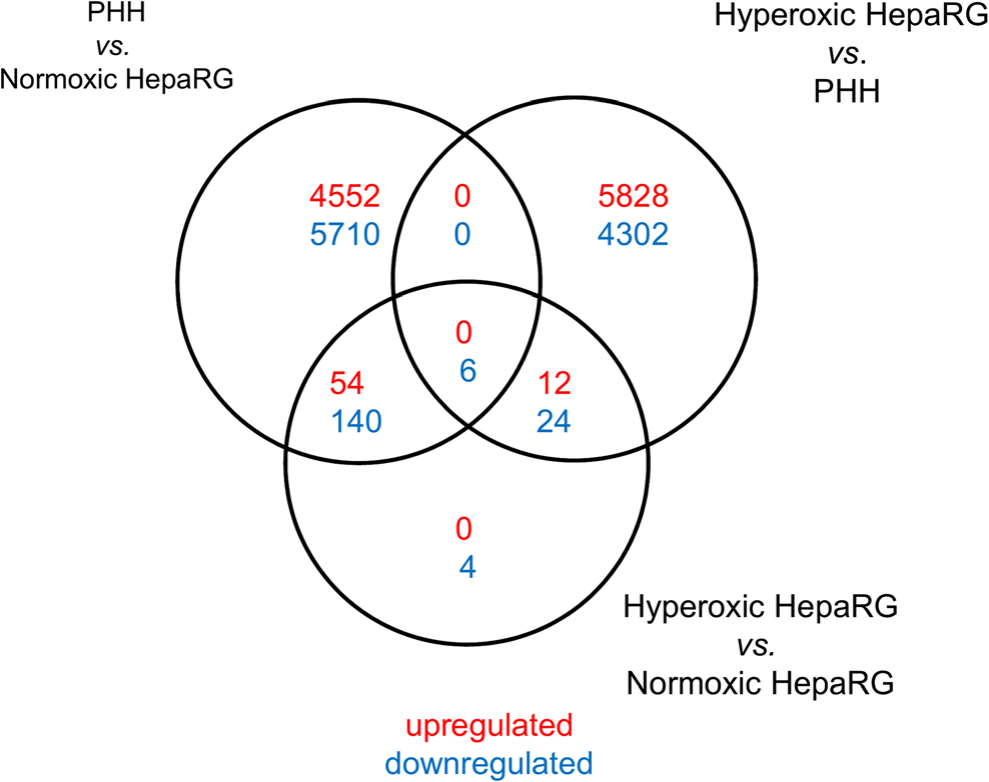
Whole-transcriptome microarray analysis on HepaRG cells cultured under normoxia or hyperoxia and primary human hepatocytes. Venn diagram of differentially expressed genes (adj. *P* < 0.05) between freshly isolated PHHs and HepaRG cells cultured under ambient normoxia or hyperoxia

To analyse the transcriptional activity underlying the increased hepatic functionality under hyperoxia, a whole-genome transcriptome analysis was performed on freshly isolated PHHs and HepaRG cells cultured under normoxia or hyperoxia. Of the 23,223 probes, 240 were differentially expressed in hyperoxic *vs* normoxic HepaRG cultures (adj. *P* < 0.05), 66 were upregulated and 174 were downregulated (Fig. 4). Of these 66 upregulated genes, 54 were also upregulated in PHHs *vs* normoxic HepaRG. The top-10 upregulated genes in hyperoxic *vs* normoxic HepaRG cells were involved in haemostasis (4/10), amino acid metabolism (2/10), signal transduction (2/10), detoxification (1/10) and carbohydrate metabolism (1/10) (Table 1). The top-10 downregulated genes were involved in extracellular matrix (ECM) and anchorage (4/10), innate immune-response (2/10) and transmembrane transport (2/10), as well as carbohydrate metabolism (1/10) and detoxification (1/10), the latter being *CYP4B1*, which is expressed in lung- rather than in liver-tissue, and is presumed inactive in humans (Schmidt et al. 2015) (Table 2).

**Table 1:** Top-10 upregulated genes in hyperoxic *versus* normoxic HepaRG cultures

Gene	Fold change	Adj. *P-*value	Protein	Function
CYP2E1	5.37	8.98∙10^−5^	Cytochrome p450 2E1	Detoxification
ASNS	3.65	9.00∙10^−3^	Asparagine Synthetase	Amino acid metabolism
F9	3.58	2.36∙10^−3^	Coagulation Factor IX	Hemostasis
PLG	3.19	3.37∙10^−4^	Plasminogen	Hemostasis
TRIB3	3.10	3.37∙10^−3^	Tribbles Pseudokinase 3	Signal transduction
PPP1R1A	3.07	2.19∙10^−3^	Protein phosphatase 1 regulatory subunit 1A	Carbohydrate metabolism
RASD1	2.67	2.19∙10^−3^	RAS, Dexamethasone-Induced 1	Signal transduction
HABP2	2.65	2.36∙10^−3^	Hyaluronan Binding Protein 2	Hemostasis
PSAT1	2.58	1.82∙10^−2^	Phosphoserine Aminotransferase 1	Amino acid metablism
FGL1	2.56	2.19∙10^−3^	Fibrinogen-Like 1	Hemostasis

**Table 2:** Top-10 downregulated genes in hyperoxic *versus* normoxic HepaRG cultures

Gene	Fold change	Adj. *P-*value	Protein	Function
COL28A1	−7.14	3.73∙10^−5^	Collagen, type XXVIII, alpha 1	Extracellular matrix
SLC10A1	−5.17	1.44∙10^−3^	Sodium/bile acid cotransporter	Transport
SPINT3	−4.96	1.60∙10^−4^	Serine Peptidase Inhibitor, Kunitz Type, 3	Extracellular matrix
KRT6A	−4.70	3.73∙10^−5^	Keratin 6 alpha	Extracellular matrix
ANKRD30A	−4.27	5.24∙10^−4^	Ankyrin repeat domain 30A	Anchorage
REG3G	−3.98	2.04∙10^−5^	Regenerating islet-derived protein 3 gamma	Innate immune response
CYP4B1	−3.79	3.92∙10^−2^	CYP4B1	Detoxification
LDHB	−3.73	2.28∙10^−4^	Lactate dehydrogenase B	Carbohydrate metabolism
FOLR1	−3.42	8.98∙10^−5^	Folate Receptor 1	Transport
PDZD3	−3.28	8.98∙10^−5^	PDZ Domain Containing 3	Innate immune response

Genes that were differentially expressed between normoxic and hyperoxic cultures were cross-referenced against the Gene Ontology transcription factor gene set (Ashburner et al. 2000), and seven transcription factors were identified (Table 3). Hepatic transcription factors One cut homeobox 2 (*ONECUT2)*, Forkhead box A3 (*FOXA3),* and *CEBPG)* were upregulated in hyperoxic *vs* normoxic cultures. The downregulated transcription factors were not known to be involved in hepatic differentiation and included Orphan nuclear receptor estrogen related receptor gamma *(ESRRG),* TSC22 domain family member 4 *(TSC22D4),* Heat-shock factor 4 *(HSF4)* and Ankyrin Repeat Domain 30A *(ANKRD30A)* .

**Table 3:** Differentially expressed genes encoding transcription factors in hyperoxic *versus* normoxic cultures

Transcription factor	Hyperoxic *versus* normoxic: Fold Change	Hyperoxic *versus* normoxic: Adj. *P*-value	PHH *versus* normoxic: Fold Change	PHH *versus* normoxic: Adj. *P*-value
CEBPG	1.79	2.26∙10^−2^	1.74	7.66∙10^−6^
FOXA3	1.97	4.998∙10^−2^	11.38	2.16∙10^−10^
ONECUT2	2.16	1.03∙10^−2^	5.36	2.40∙10^−10^
ESRRG	−2.52	8.94∙10^−3^	−6.90	9.40∙10^−8^
TSC22D3	−2.25	4.41∙10^−2^	−9.04	2.01∙10^−6^
HSF4	−2.35	1.28∙10^−3^	−1.59	1.26∙10^−2^
ANKRD30A	−4.27	5.24∙10^−4^	−6.69	9.27∙10^−9^

To further explore possible regulators of transcriptional changes, a gene signature composed of genes that were differentially expressed between normoxic HepaRG cultures and both freshly isolated PHHs and hyperoxic HepaRG cultures (non-adjusted *P* < 0.01) was analysed using Ingenuity Pathway Analysis upstream regulator analysis. Two transcription regulator target gene sets were found to overlap significantly with this gene signature: Hepatic nuclear factor 1α (*HNF1A)* was predicted to be activated (*P =* 1.14∙10^−12^), whereas V-myc avian myelocytomatosis viral oncogene homolog (*MYC)* was predicted to be inhibited *(P* = 4.45∙10^−3^) in PHH and in HepaRG under hyperoxia compared to HepaRG under normoxia. HNF1α is an established hepatic transcription factor, involved in -amongst others- bile, cholesterol, and glucose metabolism (Enosawa et al. 2006), whereas MYC is an important proto-oncogene involved in immortalization and proliferation and associated with dedifferentiation (Takahashi et al. 2007).

These data suggest that hyperoxia induces upregulation of genes that are predominantly involved in hepatic differentiation, metabolism and extracellular signalling.

### Hypoxia blocks hepatocyte differentiation of HepaRG cells

**Fig. 5:**
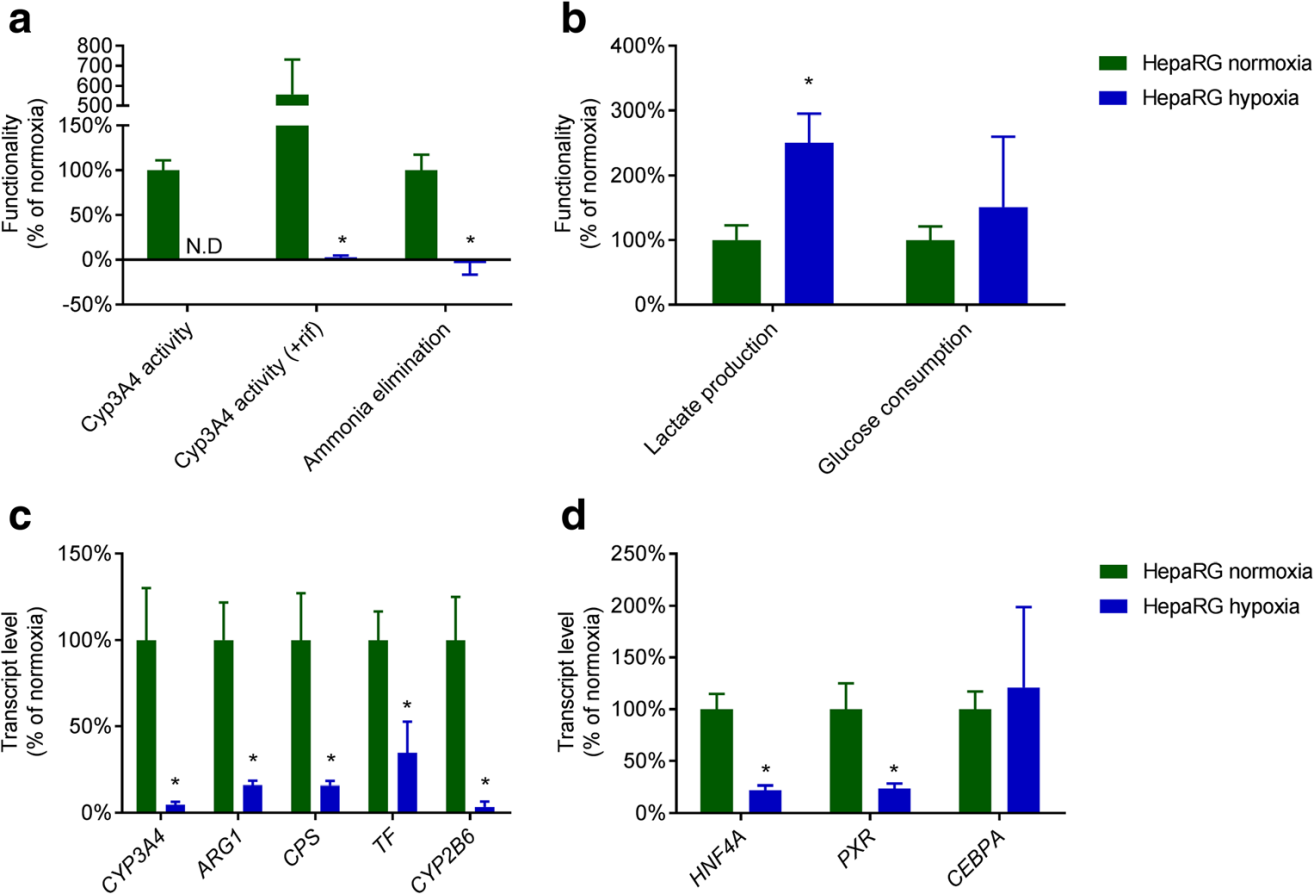
HepaRG cells cultured under hypoxia lose hepatic gene transcription and –functionality. HepaRG monolayers cultured under ambient normoxia or hypoxia were tested for hepatic functions (**a**), glucose consumption and lactate production (**b**), as well as transcript levels of hepatic genes (**c**) and transcription factor genes (**d**). * = *P* < 0.05 compared to HepaRG normoxia

Hypoxia markedly reduced hepatic functions of HepaRG cells: ammonia elimination converted into minor production, basal CYP3A4 activity was under the detection limit, and after rifampicin induction CYP3A4 activity was 0.6% of non-induced normoxic cultures (Fig. 5a). Lactate production increased up to 251%, while glucose consumption did not change significantly (Fig. 5b). Hypoxia reduced the transcript levels of all tested structural hepatic genes to 3% - 35% of the normoxic group (Fig. 5c). Transcript levels of hepatic transcription factors *HNF4A* and *PXR* were downregulated to 22% - 24% of normoxic cultures, while *CEBPA* transcript levels did not change significantly (Fig. 5d). These data confirm the opposite effects of hyperoxia and hypoxia on hepatic differentiation of HepaRG cells.

### Hypoxia-induced effects correlate with HIF1α protein expression

**Fig. 6:**
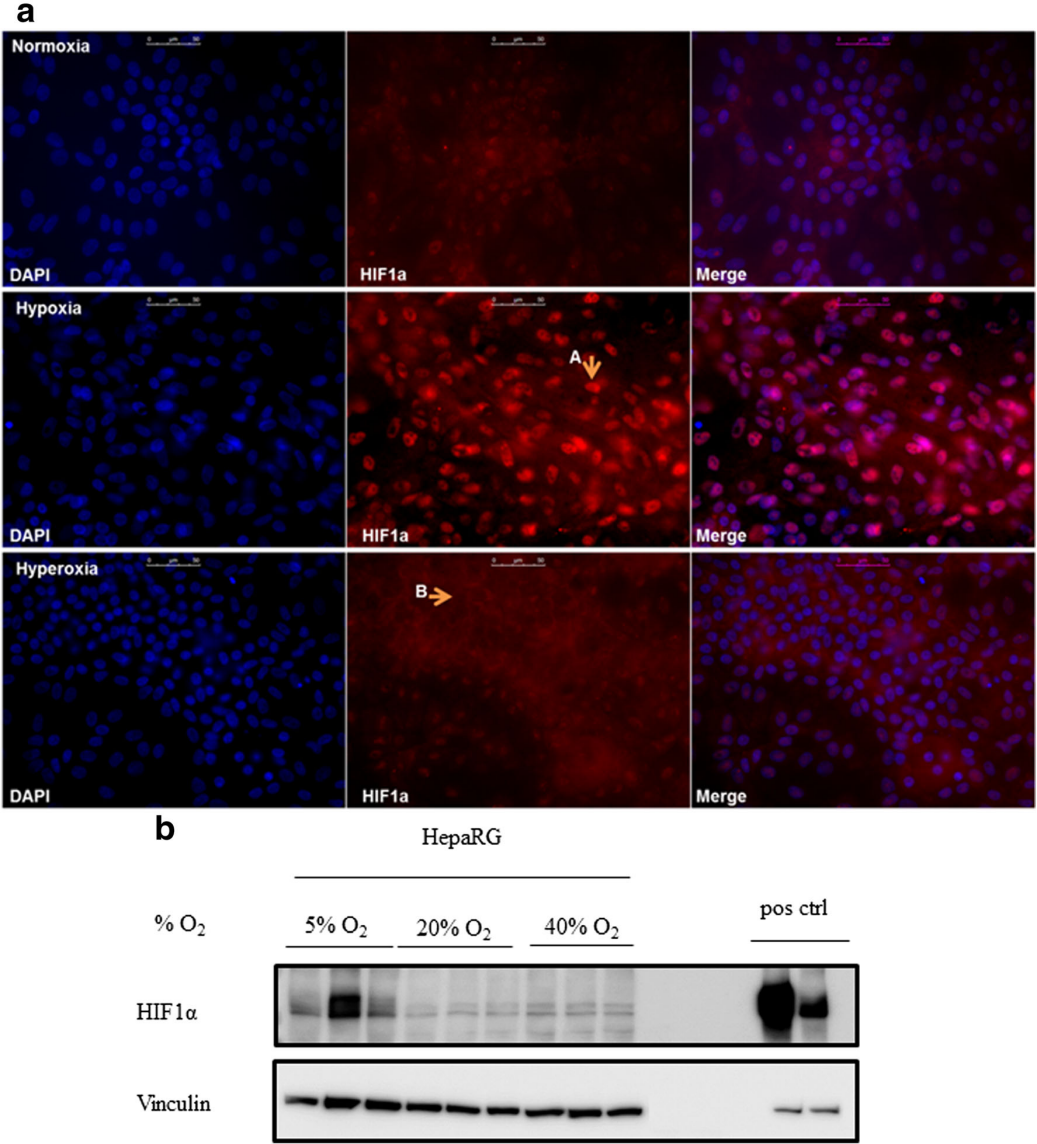
Nuclear HIF1α expression decreases at increased oxygen levels. **a** HepaRG cells cultured under normoxia (top), hypoxia (middle) and hyperoxia (bottom), stained for: DAPI (blue) and HIF1α (red). Arrow A indicates nuclear expression of HIF1α, arrow B indicates cells that appear free of nuclear HIF1α. Scale bar = 50 μM. **b** Western blot showing expression of HIF1α (top lanes), and vinculin as loading control (bottom lanes). Targets and positive controls are parts of the same image

HIF proteins are known to be important in the cellular adaptive response to oxygen. Under hypoxia, ubiquination of cytosolic HIF1α is inhibited and the protein can translocate into the nucleus and activate the transcription of hypoxia-responsive proteins (Goda and Kanai 2012). When oxygen is abundant, HIF1α is prevented to translocate to the nucleus by proteasomal degradation. Because SOX9 is a known target of HIF1α, (Amarilio et al. 2007), we hypothesized that SOX9 expression would correlate with HIF1α expression. Immunostaining revealed a clear nuclear translocation of HIF1α protein in hypoxic HepaRG cultures, while HIF1α was mainly cytosolic (and thus not able to activate transcription of hypoxia-responsive proteins) in hyperoxic cultures (Fig. 6a). In normoxic cultures, HIF1α was present in both nuclei and the cytosol. Western blotting of HepaRG samples revealed that HIF1α was most abundant in hypoxic cultures (Fig. 6b). There was no clear difference between the protein expression levels of normoxic and hyperoxic cultures.

These data show that the hypoxia induces nuclear HIF1α expression in HepaRG cells in our experimental set-up, which correlates with SOX9 expression.

### Hypoxia stabilizes HepaRG cultures

**Fig. 7:**
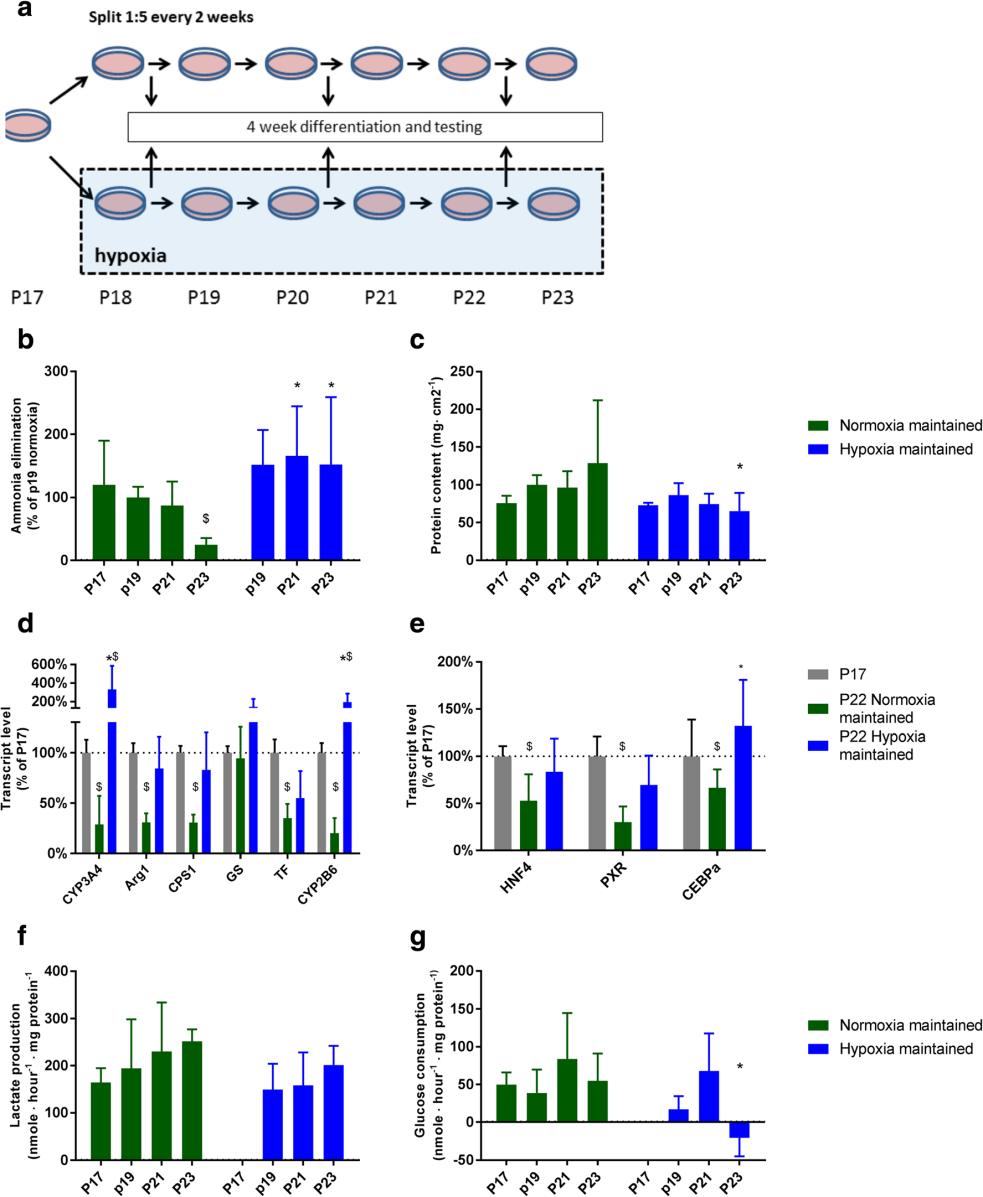
Expansion under hypoxia stabilizes the HepaRG phenotype during serial passaging. HepaRG cells at passage 17 (P17) from isolation were split into sub lines that were maintained under ambient hypoxia or normoxia. Cultures were passaged every other week, every other passage samples from both cultures were cultured under normoxia for 4 weeks and tested (**a**). Cultures were tested on ammonia elimination (**b**), total protein content (**c**), transcript levels of hepatic genes (**d**) and transcription factor genes (**e**), lactate production (**f**) and glucose consumption (**g**). * = *P* < 0.05 compared to normoxia maintained line of same passage number. $ = *P* < 0.05 compared to P17

The HepaRG cell line cannot be expanded indefinitely; after undergoing 20 passages from isolation with a split ratio of 1:5 the cells lose their capacity to fully differentiate (Laurent et al. 2013). We hypothesized that since HepaRG cells maintain their progenitor characteristics under hypoxia, this may reduce stress on the cells and improve the long-term stability. To test this, we split HepaRG lineages into two sub-lines at passage 17: one propagated under normoxia and the other one under hypoxia. Every other passage, cells from both sub-lines were cultured and differentiated under normoxia and compared head-to-head (Fig. 7a).

We confirmed that above passage 20, the normoxic-maintained lines showed a marked decrease in the hepatocyte hallmark function ammonia elimination, as well as the transcript levels of *CYP3A4, ARG1, CPS1, TF* and *CYP2B6* compared to passage 17, and that there was a trend towards an increase in total protein (*P* = 0.08 for passage 23) (Fig. 7b-e). In hypoxia-maintained lines, which were differentiated and tested under normoxia, neither ammonia elimination, total protein content nor hepatic gene transcription reduced significantly. In addition, the transcript levels of *CYP3A4* and *CYP2B6* were higher at passage 22 compared to passage 17.

We found no significant changes in lactate production over the passages and no significant difference between normoxic-maintained or hypoxic-maintained lines when differentiated an tested under normoxia (Fig. 7f). Glucose consumption was significantly lower in hypoxic-maintained sub-lines at passage 23, at which point glucose consumption turned into production (Fig. 7g).

In conclusion, these data show that maintaining HepaRG cultures under hypoxia stabilizes the capacity to hepatic differentiation under normoxia, thus increasing the total amount of HepaRG cells that can be produced from the original isolate.

## Discussion and conclusions

In this study we show that oxygen has a significant effect on the differentiation state of HepaRG cells; hypoxia promotes stem cell characteristics with increased cell line stability, whereas hyperoxia induces hepatic differentiation. In addition, hyperoxia increases the hepatic differentiation of C3A cells, which makes it highly conceivable that hyperoxia could be routinely supplied to induce hepatic differentiation in proliferative cell sources.

Hyperoxia increased all tested hepatic functions in HepaRG and C3A cells compared to normoxia-maintained cultures, and the transcript levels of most tested hepatocyte-specific genes. Whole-transcriptome analysis revealed that transcriptional changes between hyperoxic and normoxic cultures were modest, indicating a significant role of underlying post-transcriptional regulation. However, we identified several potentially contributing up- and downregulated transcription factors. Upregulated transcription factors, included *ONECUT2,* which is involved in liver cell faith and hepatoblast migration (Clotman et al. 2005; Margagliotti et al. 2007), *FOXA3*, a crucial transcription factor driving hepatocyte differentiation (Huang et al. 2014; Huang et al. 2011), and *CEBPG,* implied to stimulate oxidative phosphorylation in the liver (Shimizu et al. 2009). In addition, CEBPγ can inhibit other members of the C/EBP family through dimerization (Cooper et al. 1995). Interestingly, both *CEBPA* gene transcript levels and CEBPα nuclear protein expression were upregulated in hyperoxic cultures, indicating higher hepatocyte differentiation grade. Upregulation of *CEBPG* gene expression may therefore indicate a negative feedback loop. The most interesting downregulated transcription factors under hyperoxia were *MYC* (predicted downregulated), a proto-oncogene (Doe et al. 2012), *ESRRG,* a key regulator of hepatic gluconeogenesis (Kim et al. 2012), and *TSC22D4,* which plays a role in lipid metabolism. *TSC22D4* overexpression reduces VLDL release, while inhibition leads to hypertriglyceridemia through the induction of hepatic VLDL secretion (Jones et al. 2013).

Urea cycle activity was induced under hyperoxia, while transcription of the rate-limiting enzyme *CPS1* was not affected, indicating that the effects of hyperoxia on CPS1 are post-transcriptional.

Hypoxia kept HepaRG cells undifferentiated, as indicated by negligible hepatic functionality, low transcript levels of hepatic genes and nuclear expression of progenitor marker SOX9 (Huch et al. 2015), which is in line with previous observations that hypoxia is beneficial for the development of hepatic progenitor cells from embryonic stem cell-derived endoderm (Katsuda et al. 2013). SOX9 has been described to induce hepatocyte dedifferentiation, while CEBPα can counteract this effect, resulting in both transcription factors functioning as reciprocal repressors (O'Neill et al. 2014).

Hypoxia induced HIF1α expression and nuclear translocation in HepaRG cultures, which likely accounted for the observed HIF-associated effects, including the nuclear translocation of SOX9, the increased lactate production, and maintenance of stem cell characteristics. A vast range of effects have been attributed to HIFs, as an adaptation of mitochondrial respiration to hypoxia, amongst which the downregulation of free fatty acid synthesis (mainly HIF2α), stimulation of anaerobic glycolysis and inhibition of pyruvate dehydrogenase (mainly HIF1α) (Goda and Kanai 2012). These may, at least in part be modulated by MYC, as MYC-target gene transcription negatively correlated with oxygenation grade. In addition to HIFs, MYC is reported to be upregulated under hypoxia and is also a downstream target of HIFs. However, the interplay with HIFs is complex, as HIF may also repress MYC activity during small and severe hypoxic events (Zwaans and Lombard 2014; Ham 3rd and Raju 2016). In addition to HIFs, other less-studied mechanisms may play a role, such as activation of the Raf-ERK pathway by NDR3-stabilization through increased lactate levels (Lee et al. 2015).

The pericellular oxygen concentration in both normoxic (181 ± 2.4 μM) and hyperoxic cultures (339 ± 3.9 μM) in this study is beyond the physiological concentration that ranges between 65 μM periportally and 35 μM pericentrally (Jungermann and Kietzmann 2000; Martinez et al. 2008). It has been described that primary mouse hepatocytes retain their functionality longer after isolation under 40% ambient oxygen, and that oxygen consumption was three-fold higher *in vitro vs in vivo (Buck et al.* 2014*)*. Also, primary porcine hepatocytes cultured in bioartificial livers under medium perfusion showed higher functionality & stability at 250 μM (40%O_2_) *vs* 130 μM (20% O_2_). Clearly, the optimal oxygen concentration *in vitro* depends on the developmental state of the cells; as HepaRG cells lost viability under hyperoxia when applied in the proliferation phase, and not when applied in the differentiation phase (Cerec et al. 2007). In addition, propagation of the HepaRG cells was optimal under hypoxia, while differentiation was clearly inhibited by low oxygen concentration. This indicates that oxygen concentration has to be optimized for each developmental stage in a culture-protocol.

The experiments in this paper were all performed in tumour-derived cell lines. Primary hepatocytes are not a suitable model, since their phenotypes are not stable *in vitro* (Meyer et al. 2013) and increase in oxygen tension does not revert this phenomenon (Poyck et al. 2008). Although stem cell derived hepatocyte-like cells hold great promise, differentiated HepaRG are the closest representation of human hepatocytes available at the moment (van Wenum et al. 2014; Gao and Liu 2017). Both the total supply of HepaRG cells and –relevant for therapeutic application- batch sizes are limited by the maximum number of passages. Increase in the expansion potential provides a significant benefit to the development of new cell-based medicinal therapies.

In this manuscript we describe that the hepatocyte differentiation grade of HepaRG and C3A cells is influenced by oxygenation grade, underlining the plasticity of these cells. Here we did not study reversibility of the hyperoxia effect, although we believe it is highly likely that hepatic markers will reduce upon reduction of oxygen concentration until normoxia or hypoxia. In a recent study, we show that even primary hepatocytes lose their phenotype under hypoxia, while it is maintained under hyperoxia (Giglioni et al., Hepatology Communications, in press).

In conclusion, we show that oxygen is a driving factor in hepatocyte differentiation in hepatocyte cell lines, and that higher levels of oxygen correspond to lower nuclear expression of SOX9 and HIF1α, highlighting the importance of adjusted pericellular oxygen tension to the development stage of *in vitro* liver cell cultures. In addition, we show that hypoxia improves the propagation capacity of HepaRG cells.

## Electronic supplementary material

Figure S1. **Negative controls for immunofluorescent stainings.** Negative controls (secondary antibody only), performed during the same experiments and taken at the same settings as the stainings in Fig 2A (**A.** DAPI, Albumin, SOX9, and merge) Fig 2B (**B.** DAPI, CEBPα, and merge) and Fig 6A (**C** DAPI, HIF1α, and merge). (GIF 75 kb)

(GIF 74 kb)

(GIF 90 kb)

High Resolution Image (TIFF 211 kb)

High Resolution Image (TIFF 265 kb)

High Resolution Image (TIFF 276 kb)

Table S1 (DOC 33 kb)
