# Supplementary figures and images for: Oxygen drives hepatocyte differentiation and phenotype stability in liver cell lines
Source: J Cell Commun Signal. 2018 Feb 4;12(3):575–88. doi: 10.1007/s12079-018-0456-4 (PMC6039343; doi:10.1007/s12079-018-0456-4)

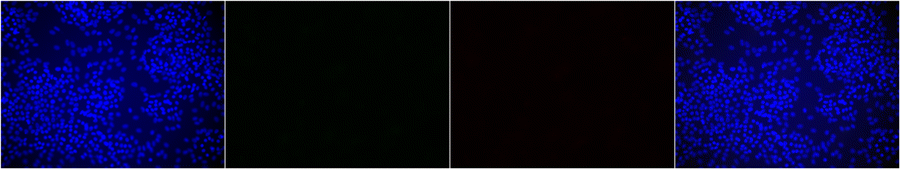

Supplement: Supplementary file 1 — Negative controls for immunofluorescent stainings. Negative controls (secondary antibody only), performed during the same experiments and taken at the same settings as the stainings in Fig 2A (A. DAPI, Albumin, SOX9, and merge) Fig 2B (B. DAPI, CEBPα, and merge) and Fig 6A (C DAPI, HIF1α, and merge). (GIF 75 kb) [file 12079_2018_456_Fig8_ESM.gif]

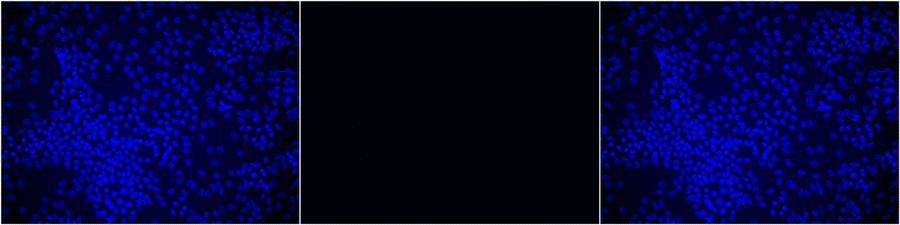

Supplement: Supplementary file 2 — (GIF 74 kb) [file 12079_2018_456_Fig9_ESM.gif]

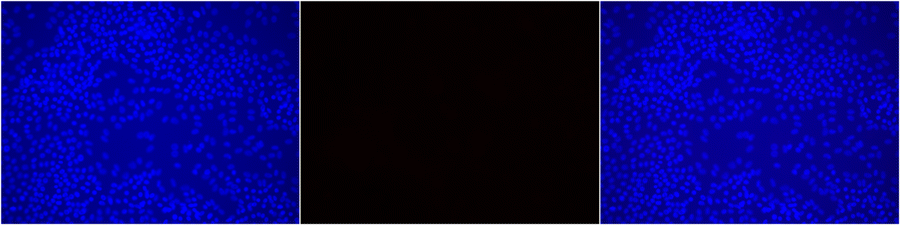

Supplement: Supplementary file 3 — (GIF 90 kb) [file 12079_2018_456_Fig10_ESM.gif]

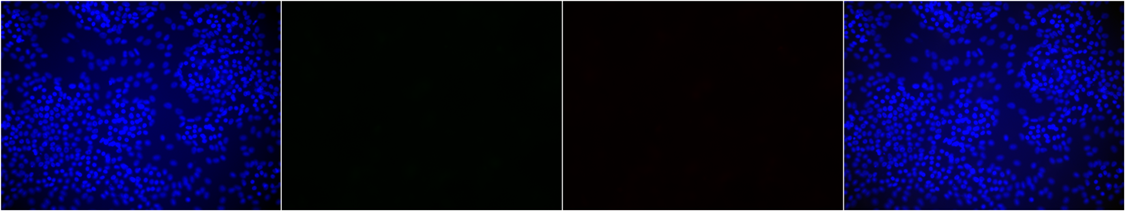

Supplement: Supplementary file 4 — High Resolution Image (TIFF 211 kb) [file 12079_2018_456_MOESM1_ESM.tif]

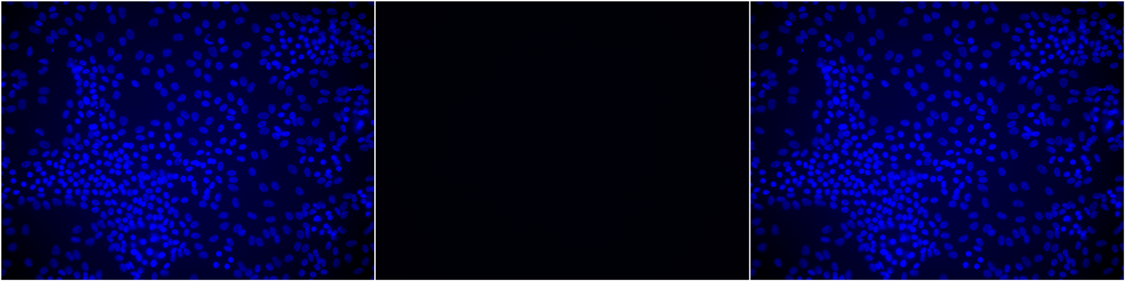

Supplement: Supplementary file 5 — High Resolution Image (TIFF 265 kb) [file 12079_2018_456_MOESM2_ESM.tif]

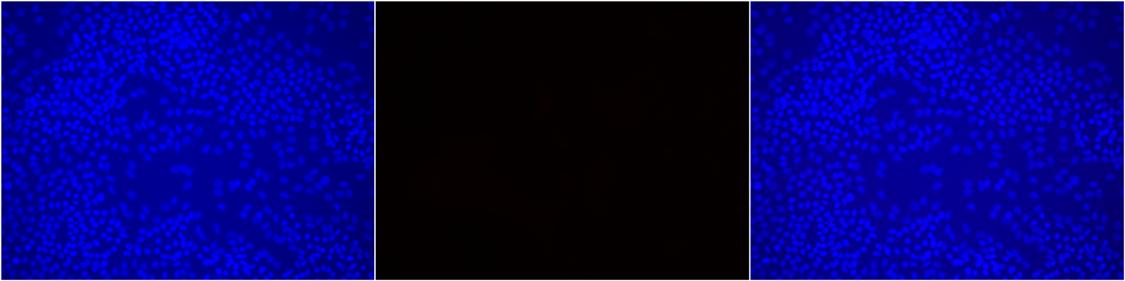

Supplement: Supplementary file 6 — High Resolution Image (TIFF 276 kb) [file 12079_2018_456_MOESM3_ESM.tif]
