# Supplementary material for: Oxygen drives hepatocyte differentiation and phenotype stability in liver cell lines
Source: J Cell Commun Signal. 2018 Feb 4;12(3):575–88. doi: 10.1007/s12079-018-0456-4 (PMC6039343; doi:10.1007/s12079-018-0456-4)
Supplement: Supplementary file 7 — (DOC 33 kb) [file 12079_2018_456_MOESM4_ESM.doc]

**Table S1.**

Primer sequences used in the RT-PCR analyses with resulting amplicon sizes. Primers are indicated from 5’  3’.

| Gene | Sense primer | Antisense primer | Amplicon size (bp) |
| --- | --- | --- | --- |
| *18S rRNA* | TTCGGAACTGAGGCCATGAT | CGAACCTCCGACTTTCGTTCT | 151 |
| *ARG1* | TTGGCAAGGTGATGGAAGAAACA | CCTCCCGAGCAAGTCCGAAACAA | 305 |
| *CAR* | CGTCATGGCCAGTAGGGAAG | CATGCCAGCATCTAAGCACT | 232 |
| *CEBPA* | GTGCGTCTAAGATGAGGGGG | GGAAGGAGGCAGGAAACCTC | 203 |
| *CPS* | GAAGGGGCCCGAGAAGTAGAA | CTCAACCGGGGCCAGGAAAAC | 445 |
| *CYP2B6* | CCCGCCCTCTGCCCCTTTTG | TCCACACTCCGCTTTCCCATCC | 328 |
| *CYP3A4* | AGCTTAGGAGGACTTCTTCAACC | AGCCAAATCTACCTCCTCACACT | 313 |
| *GS* | GCCTGCTTGTATGCTGGAGTC | GGCGCTACGATTGGCTACAC | 420 |
| *HNF4A* | CCGGGTGTCCATACGCATCCT | CAGGTTGTCAATCTTGGCC | 321 |
| *PGC1* | CAGGTGCCTTCAGTTCACTCT | AACCAGAGCAGCACACTCGAT | 181 |
| *PXR* | GAGAGCGGCATGAAGAAGGAGA | CATGTGGGGCAGCAGGGAGAAG | 420 |
| *TF* | GAAGGACCTGCTGTTTAAGG | CTCCATCCAAGCTCATGGC | 310 |
